# Supplementary material for: Realizing thermoelectric cooling and power generation in N-type PbS0.6Se0.4 via lattice plainification and interstitial doping
Source: Nat Commun. 2024 May 6;15:3782. doi: 10.1038/s41467-024-48268-3 (PMC11074254; doi:10.1038/s41467-024-48268-3)
Supplement: Supplementary file 1 — Supplementary Information [file 41467_2024_48268_MOESM1_ESM.pdf]

Supplementary Information

Lei Wang[1], Yi Wen[1], Shulin Bai[1,2], Cheng Chang[1], Yichen Li[1], Shan Liu[1], Dongrui Liu[1], Siqi Wang[1], Zhe Zhao[1], Shaoping Zhan[1], Qian Cao[3], Xiang Gao[4], Hongyao Xie[1,*], Li-Dong Zhao[1,2,*]

[1] *School of Materials Science and Engineering, Beihang University, Beijing 100191, China.*

[2] *Tianmushan Laboratory, Yuhang District, Hangzhou 311115, China.*

[3] *Huabei Cooling Device Co. LTD., Hebei 065400, China.*

[4] *Center for High Pressure Science and Technology Advanced Research (HPSTAR), Beijing 100094, China.*

E-mail: xiehongyao@buaa.edu.cn; *zhaolidong@buaa.edu.cn*

Lorenz number calculation:

The Lorenz number (L) is used to evaluate the electronic thermal conductivity (κ_{ele}) with a relationship of $\kappa_{ele} = L\sigma T$, where the σ is the electrical conductivity, T denotes working temperature. The Lorenz number can be obtained by fitting the Seebeck coefficient to the reduced chemical potential with following equations:

$$L=\left(\frac{k_B}{e}\right)^2\left(\frac{\left(r+\frac{7}{2}\right)F_{r+\frac{5}{2}}(\eta)}{\left(r+\frac{3}{2}\right)F_{r+\frac{1}{2}}(\eta)}-\left[\frac{\left(r+\frac{5}{2}\right)F_{r+\frac{3}{2}}(\eta)}{\left(r+\frac{3}{2}\right)F_{r+\frac{1}{2}}(\eta)}\right]^2\right) \tag{1}$$

where k_B is the Boltzmann constant, e is the electric charge, r is the scattering parameter, and η refers to the reduced Fermi energy, which can be derived from the measured Seebeck coefficients with consideration of acoustic phonon dominated scattering (r = -1/2)

$$S=\frac{k_B}{e}\left[\frac{\left(r+\frac{5}{2}\right)F_{r+\frac{3}{2}}(\eta)}{\left(r+\frac{3}{2}\right)F_{r+\frac{1}{2}}(\eta)}-\eta\right] \tag{2}$$

where the $F_n(\eta)$ is the n-th order Fermi integral:

$$Fx(\eta)=\int_0^{+\infty}\frac{\varepsilon^x}{1+exp(\varepsilon-\mu)}d\varepsilon \tag{3}$$

Weighted mobility calculation:

Weighted mobility is calculated through the following equation:[1]

$$\mu_W=\frac{3h^3\sigma}{8\pi e(2m_e k_B T)^{3/2}}\left[\frac{\exp\left[\frac{|S|}{k_B/e}-2\right]}{1+\exp\left[-5\left(\frac{|S|}{k_B/e}-1\right)\right]}+\frac{\frac{3}{\pi^2}\frac{|S|}{k_B/e}}{1+\exp\left[5\left(\frac{|S|}{k_B/e}-1\right)\right]}\right] \tag{4}$$

where μ_W is weighted mobility, h is Planck constant, m_e is the mass of electron, k_B is the Boltzmann constant, e is the electric charge.

First-principles Calculation:

First-principles calculations with projected augmented wave (PAW) pseudopotential formalism were performed within the Perdew-Burke-Ernzerhof (PBE) exchange-correlation functional form of generalized gradient approximation (GGA) method as implemented in Vienna Ab-initio Simulation Package (VASP) software[2-4]. The supercell of $Pb_{27}S_{16}Se_{11}$ and $Pb_{27}S_{16}Se_{11}Cu$ (Cu was

placed in the interstitial position) were constructed to perform projected density of states calculations. The wave functions were adopted in plane wave basis with the kinetic energy cut-off of 550 eV, and the Monkhorst-Pack k-meshes of 5×5×5 was adopted in this work.

For conduct defect calculations, a 4×4×4 supercell containing 128 atoms for the primitive cell of PbS structure ($Pb_{64}S_{64}$) was performed in this work, and the Monkhorst-Pack k-meshes of 4×4×4 was used by the conjugated gradient method to sample in the Brillouin Zone. The wave functions were adopted in plane wave basis with the kinetic energy cut-off of 550 eV. Due to the presence of heavy Pb element, the spin-orbital coupling effect[5] was also separately considered in our calculations, which would affect the bandgaps and the positions of the band edges. The convergence criterions for the total energy and Hellmann-Feynman force were less than 10^{-6} eV and 10^{-2} eV/Å, respectively. The lattice constant and ion position of the perfect supercell were relaxed, while for the supercells containing defects, the cell volumes were kept constant and the ion positions were relaxed so as to meet the dilute limit condition[6,7].

The stability of a defect was determined by its formation energy, which is defined as[7]

$$\Delta H_{d,q} = E_{d,q} - E_{pure} - \sum_{i} n_i(E_i - \mu_i) + q(E_V + E_F + \Delta V) \quad (5)$$

where $\Delta H_{d,q}$ represents the formation energy of a defect (d) in charge state (q). $E_{d,q}$ and E_{pure} are the total energy of the defect system and perfect supercell, respectively. n_i and E_i are the number and total energy of the i-th type (host atoms or impurity atoms) added to ($n_i > 0$) or taken from ($n_i < 0$) the supercells in order to create the defect. μ_i refers the corresponding chemical potentials of these atoms, which usually depends on experiment conditions. For a maximally rich growth environment of an element i, $\mu_i = 0$. q is the corresponding charge value of a charged defect. E_F is the Fermi level with respect to E_V, which is the valence band maximum (VBM) of the perfect supercell. ΔV indicates the electrostatic potential difference between the perfect supercell and defective system, which is aligned with the corresponding VBM. The difference of the average electrostatic potential with the atoms far away from the defect sites was adopted in this work.

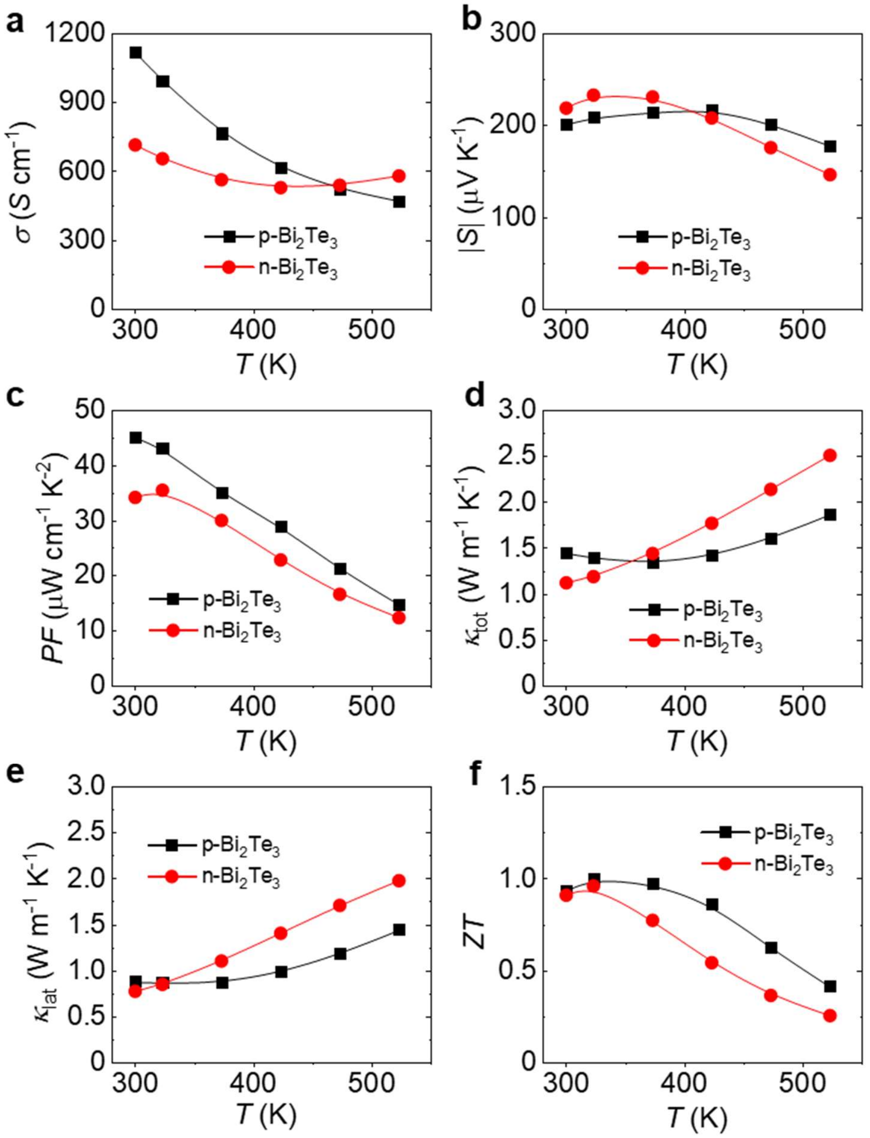

Supplementary Fig. 1. Transport properties of n-type and p-type commercial Bi_2Te_3. Temperature-dependent **a** electric conductivity, **b** Seebeck coefficient, **c** power factor, **d** total thermal conductivity, **e** lattice thermal conductivity, **f** *ZT* of commercial n-type Bi_2Te_3 for comparison with our optimal sample and p-type Bi_2Te_3 sample used to construct thermoelectric module.

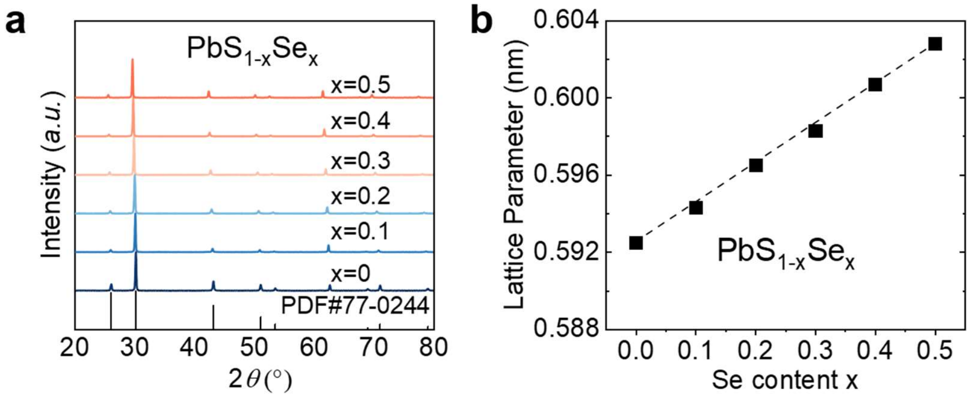

Supplementary Fig. 2. Microstructure properties of polycrystalline $PbS_{1-x}Se_x$ samples. a Powder XRD pattern of $PbS_{1-x}Se_x$ ($x = 0 - 0.5$) samples and **b** lattice parameter of the corresponding samples.

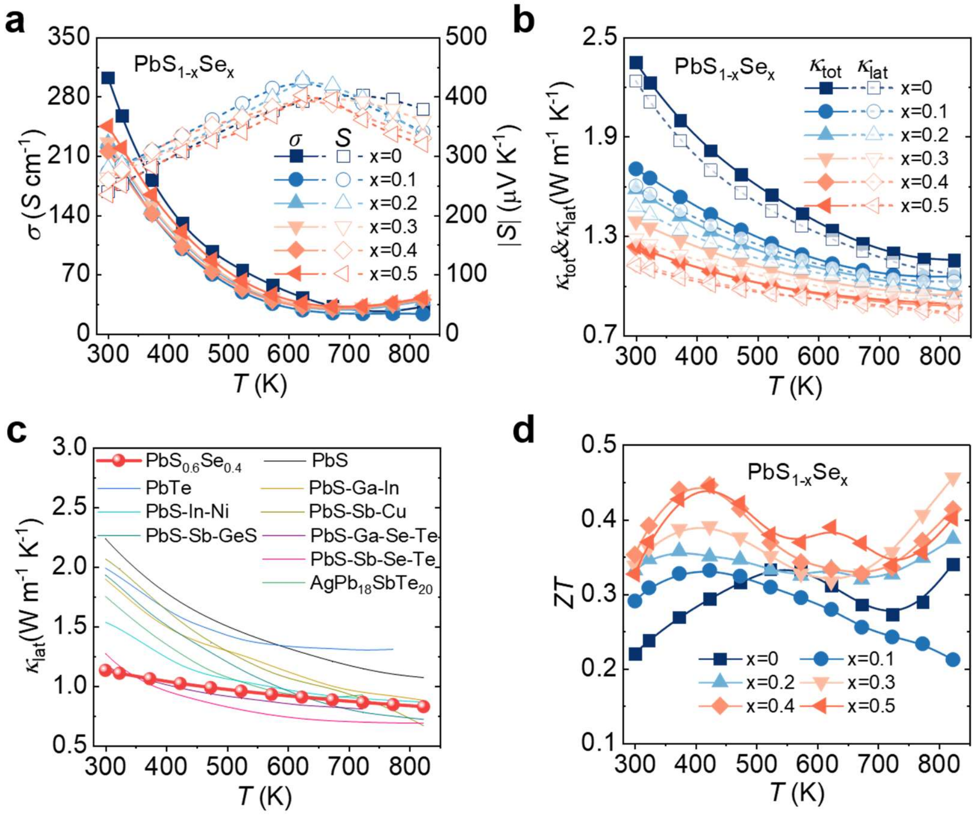

Supplementary Fig. 3. Transport properties of polycrystalline $PbS_{1-x}Se_x$ samples. Temperature dependence of **a** electrical conductivity and Seebeck coefficient, **b** total thermal conductivity and lattice thermal conductivity of $PbS_{1-x}Se_x$ samples, **c** the comparison of lattice thermal conductivity for our $PbS_{0.6}Se_{0.4}$ and other reported lead chalcogenide,[8-15] **d** *ZT* value of $PbS_{1-x}Se_x$ (x = 0 – 0.5) samples.

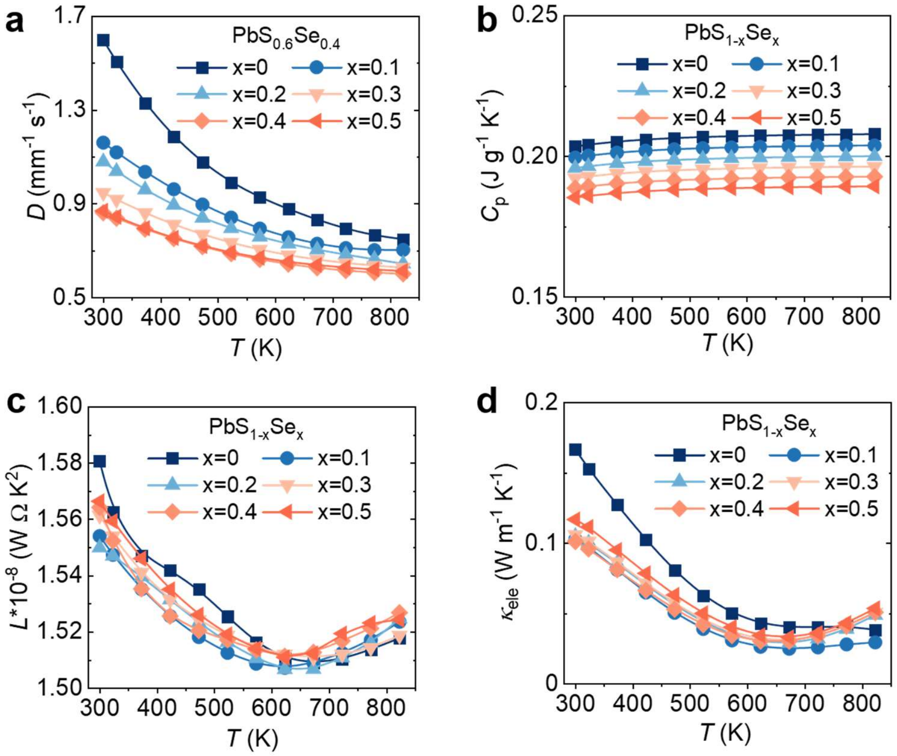

Supplementary Fig. 4. Transport relevant parameters of polycrystalline $PbS_{1-x}Se_x$ samples. Temperature-dependent **a** thermal diffusivity, **b** heat capacity, **c** Lorenz number, **d** electronic thermal conductivity of $PbS_{1-x}Se_x$ (x = 0 – 0.5) samples.

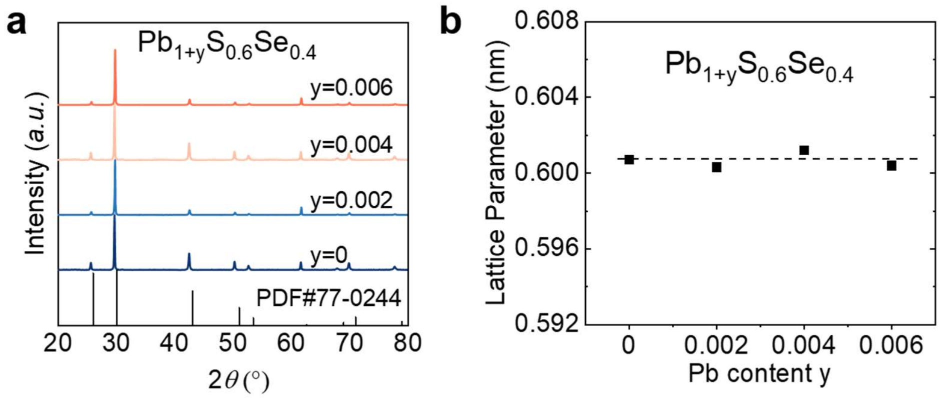

Supplementary Fig. 5. Microstructure properties of polycrystalline $Pb_{1+y}S_{0.6}Se_{0.4}$ samples. a Powder XRD pattern of $Pb_{1+y}S_{0.6}Se_{0.4}$ (y = 0 - 0.006) samples and **b** lattice parameter of the corresponding samples.

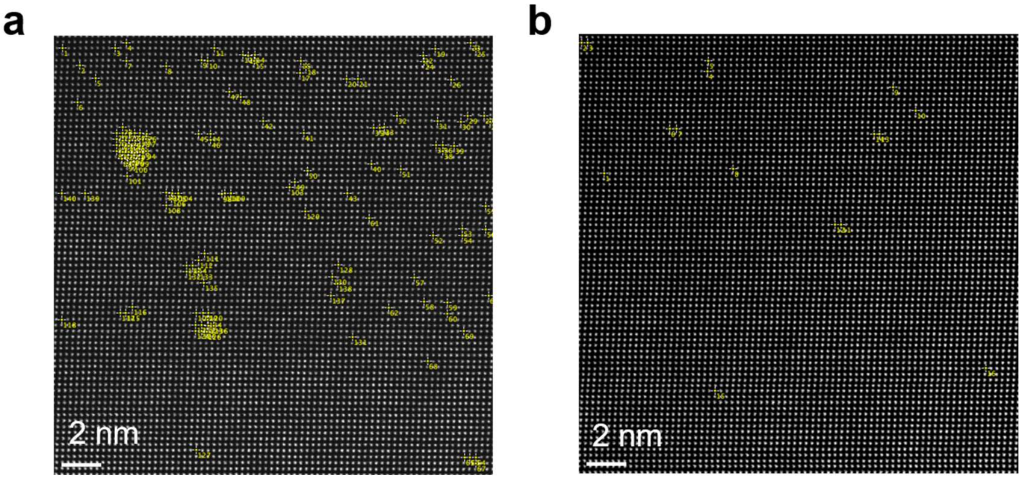

Supplementary Fig. 6. Vacancies counting results for $PbS_{0.6}Se_{0.4}$ and $Pb_{1.004}S_{0.6}Se_{0.4}$ samples. Counting of vacancies in **a** $PbS_{0.6}Se_{0.4}$ and **b** $Pb_{1.004}S_{0.6}Se_{0.4}$, respectively, both imaged along [100]. Before Pb addition, the density of vacancy was 0.28 nm^{-2}. After Pb addition, the density of vacancy was 0.03 nm^{-2}.

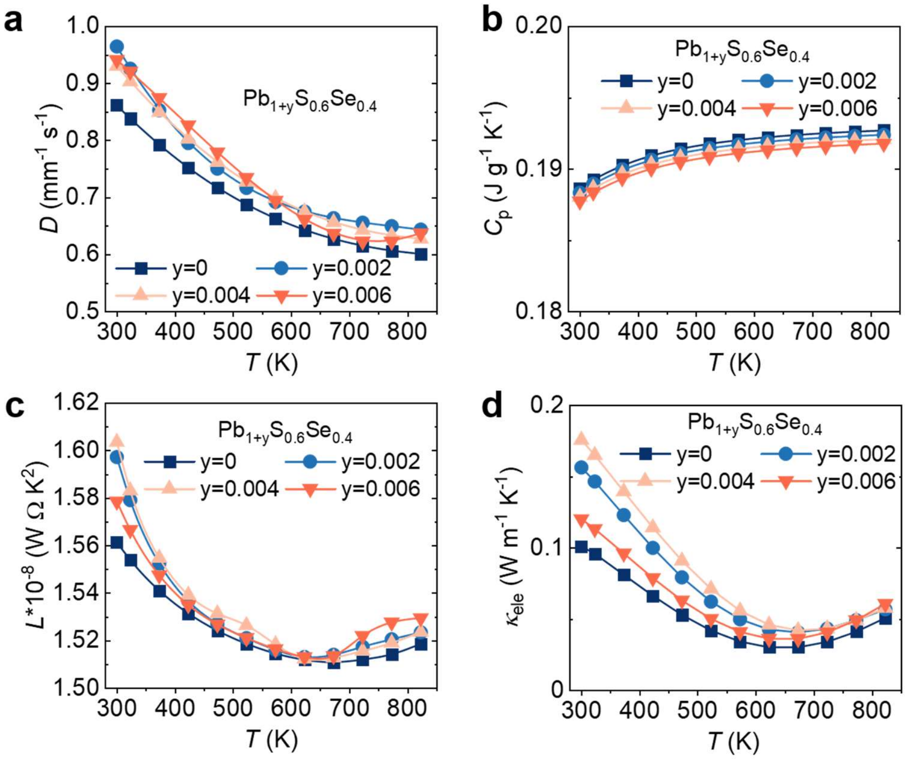

Supplementary Fig. 7. Transport relevant parameters of polycrystalline $Pb_{1+y}S_{0.6}Se_{0.4}$ samples. Temperature-dependent **a** thermal diffusivity, **b** heat capacity, **c** Lorenz number, **d** electronic thermal conductivity of $Pb_{1+y}S_{0.6}Se_{0.4}$ (y = 0 – 0.006) samples

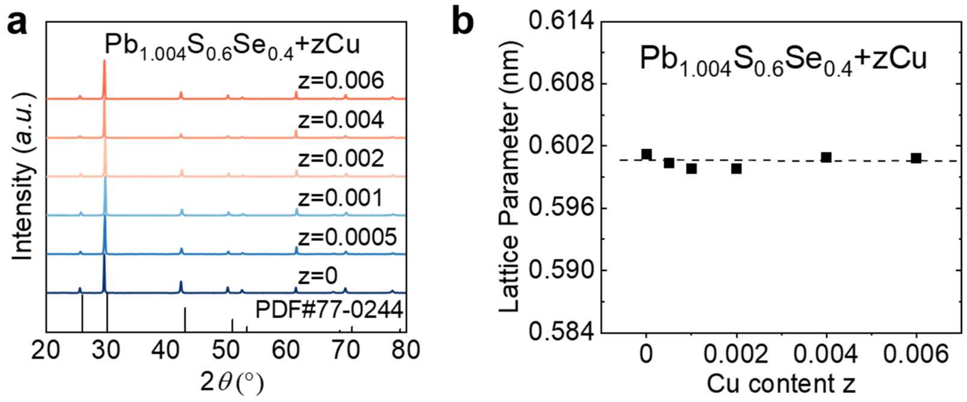

Supplementary Fig. 8. Microstructure properties of polycrystalline $Pb_{1.004}S_{0.6}Se_{0.4}$ + zCu samples. **a** Powder XRD pattern of $Pb_{1.004}S_{0.6}Se_{0.4}$ + zCu (z = 0 - 0.006) samples and **b** lattice parameter of the corresponding samples.

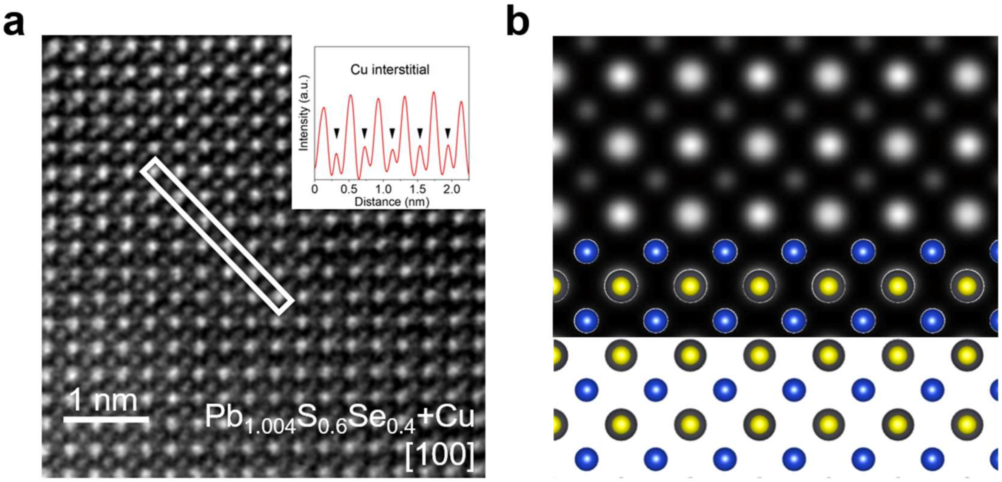

Supplementary Fig. 9. Experimental observation and simulation for occupancy of Cu atoms in polycrystalline $Pb_{1.004}S_{0.6}Se_{0.4}$ + 0.001Cu sample. a Line intensity scan profile of $Pb_{1.004}S_{0.6}Se_{0.4}$ + 0.001Cu sample; **b** multislice simulation (performed using QSTEM code[16,17] and imaging parameters of experimental data acquisition) of ADF-STEM image based on crystal model showing Cu interstitial atoms (blue spheres) in PbS matrix (Pb - gray spheres, S - yellow spheres).

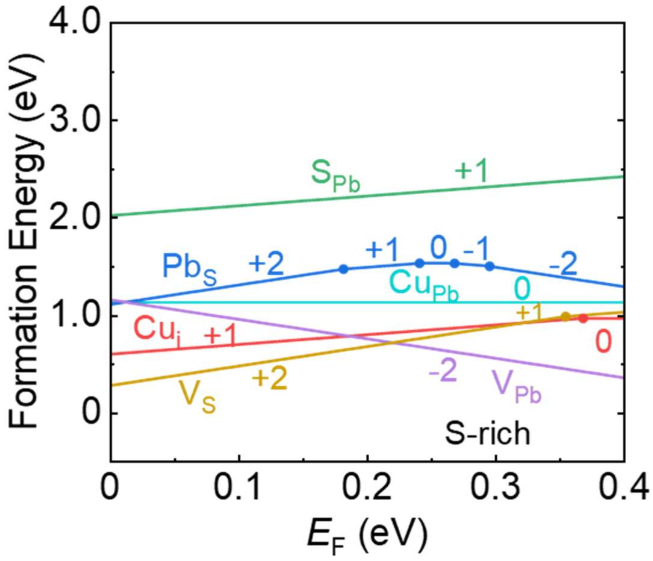

Supplementary Fig. 10. The formation energy calculation results for point defects in PbS as the function of Fermi energy (E_F) under Se-rich condition.

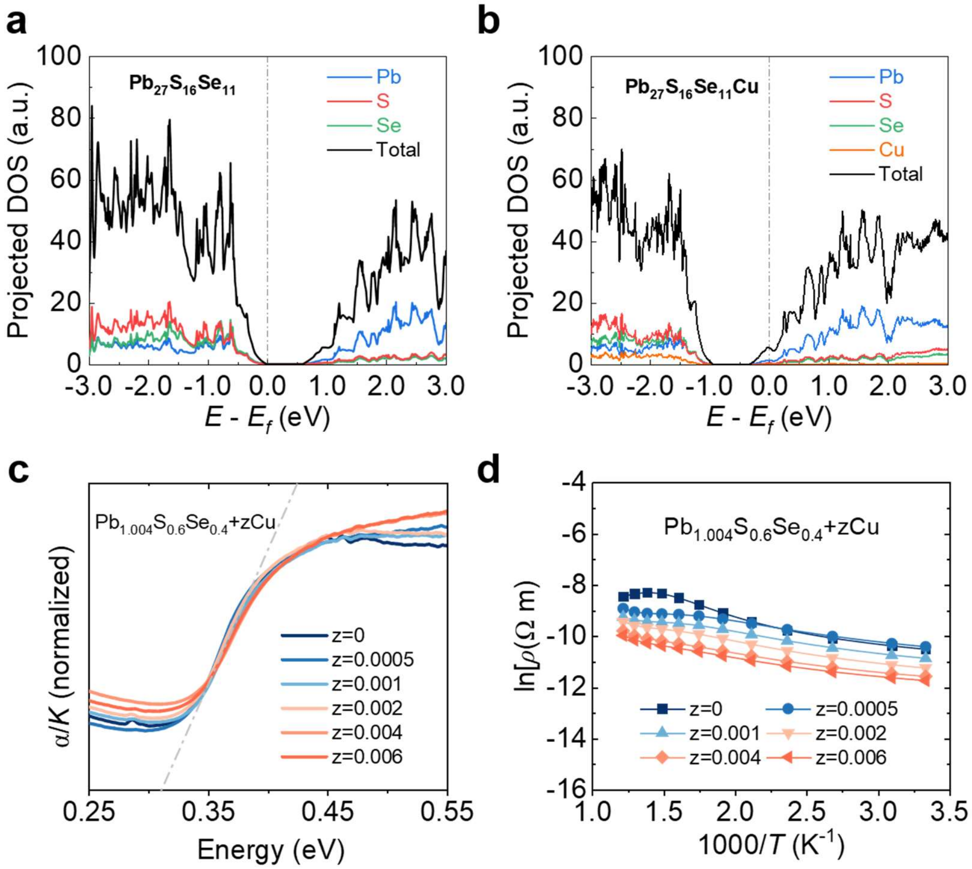

Supplementary Fig. 11. Calculated and experimental band gap results of polycrystalline $Pb_{1.004}S_{0.6}Se_{0.4}$ + zCu samples. The projected density of states (DOS) of **a** $Pb_{27}S_{16}Se_{11}$ and **b** $Pb_{27}S_{16}Se_{11}Cu$. The calculated band gap results are already the same. **c** Optical band gap test results (0.31eV) measured by Fourier Transform Infrared Spectrometer (IRAffinity-1S, SHIMADZU) and **d** lnρ - T^{-1} of $Pb_{1.004}S_{0.6}Se_{0.4}$ + zCu (z = 0 – 0.006) samples. **d** indicates the material exhibit heavily doped semiconductor transport characteristics and have similar slope, which means the energy band gap are basically unchanged.

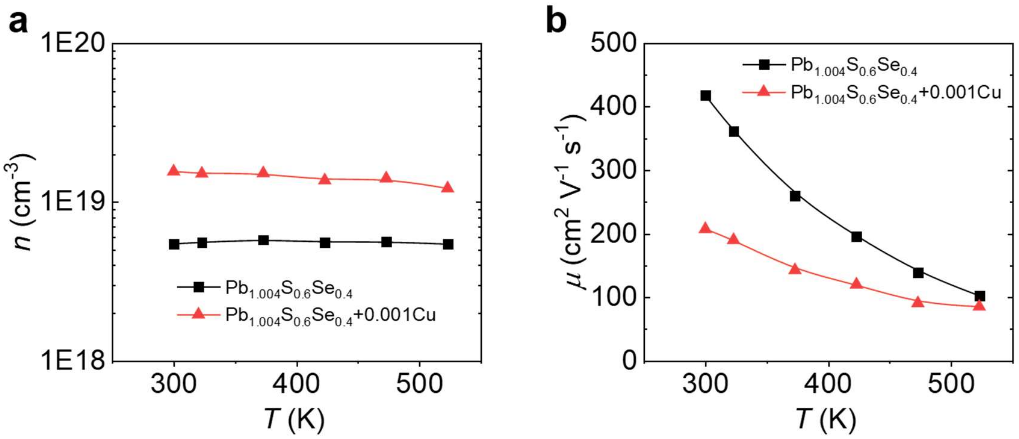

Supplementary Fig. 12. Carrier transport properties of polycrystalline $Pb_{1.004}S_{0.6}Se_{0.4}$ and $Pb_{1.004}S_{0.6}Se_{0.4}$ + 0.001Cu samples. The temperature dependent **a** carrier concentration and **b** carrier mobility of $Pb_{1.004}S_{0.6}Se_{0.4}$ and $Pb_{1.004}S_{0.6}Se_{0.4}$ + 0.001Cu samples. The carrier concentration of all samples does not change with increasing temperature in the observed temperature range, and their carrier mobility significantly decrease with rising temperature, exhibiting the degenerated semiconductor behavior.

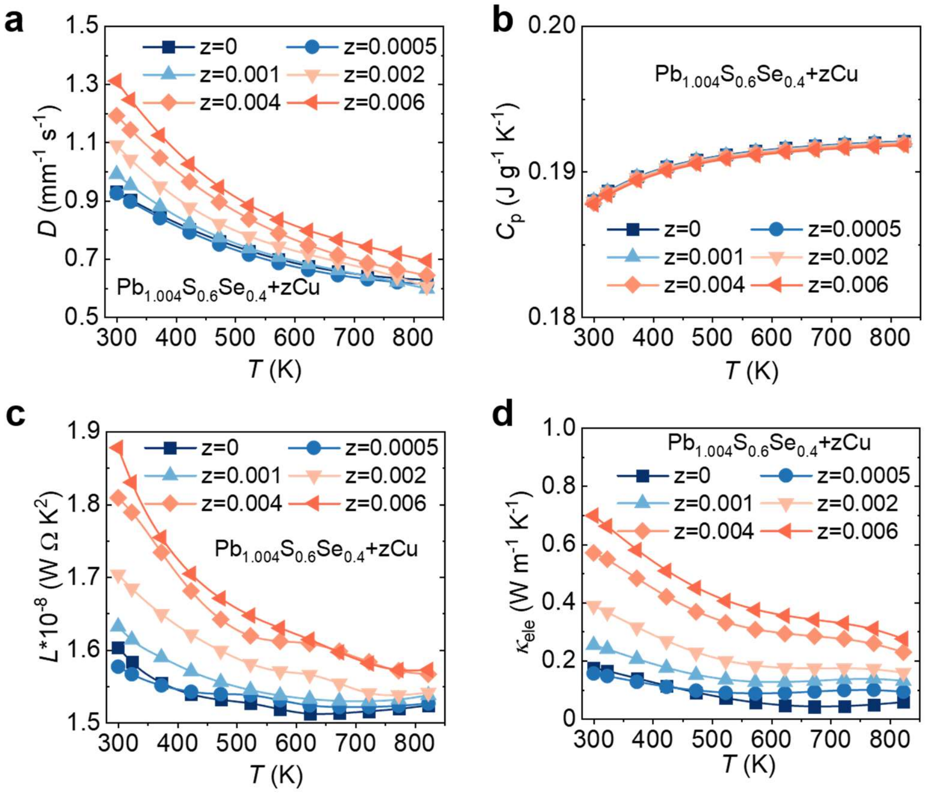

Supplementary Fig. 13. Transport relevant parameters of polycrystalline $Pb_{1.004}S_{0.6}Se_{0.4}$ + zCu samples. Temperature-dependent **a** thermal diffusivity, **b** heat capacity, **c** Lorenz number, **d** electronic thermal conductivity of $Pb_{1.004}S_{0.6}Se_{0.4}$ + zCu (z = 0 – 0.006) samples.

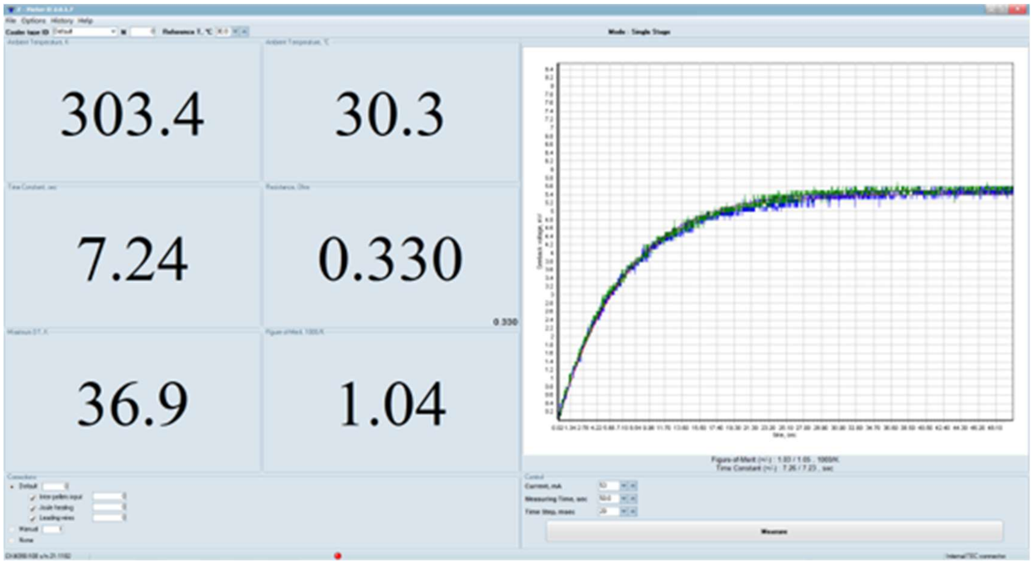

Supplementary Fig. 14. The result of refrigeration temperature difference tested by *Z*-meter.

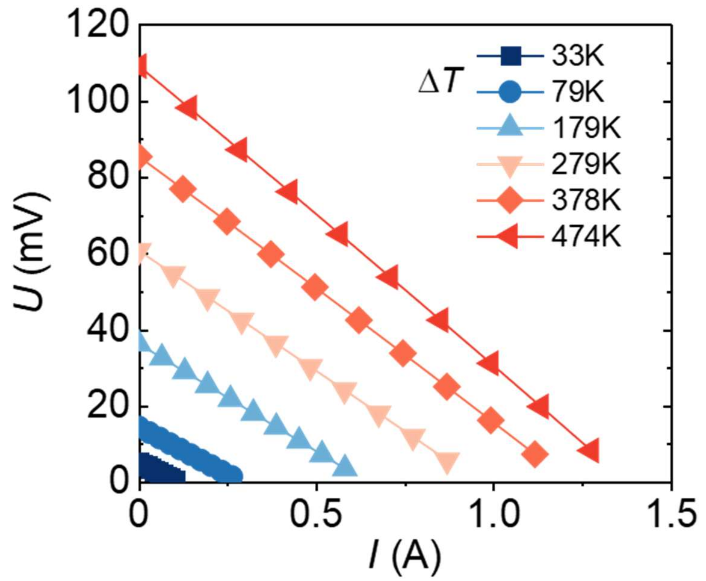

Supplementary Fig. 15. The experimentally measured output Voltage U with respect to electric current I of the $Pb_{1.004}Se_{0.6}S_{0.4}$ + 0.001 Cu crystal sample

Supplementary References

1. Snyder, G. J. *et al.* Weighted Mobility. *Adv. Mater.* **32**, 2001537 (2020).
2. Hafner, J. Ab-initio simulations of materials using VASP: Density-functional theory and beyond. *J. Comput. Chem.* **29**, 2044-2078 (2008).
3. Perdew, J. P., Burke, K. & Ernzerhof, M. Generalized Gradient Approximation Made Simple. *Phys. Rev. Lett.* **77**, 3865-3868 (1996).
4. Blöchl, P. E. Projector augmented-wave method. *Phys. Rev. B* **50**, 17953-17979 (1994).
5. Filatov, M. & Cremer, D. Calculation of indirect nuclear spin–spin coupling constants within the regular approximation for relativistic effects. *J. Chem. Phys.* **120**, 11407-11422 (2004).
6. Vidal, J. *et al.* Band-structure, optical properties, and defect physics of the photovoltaic semiconductor SnS. *Appl. Phys. Lett.* **100** (2012).
7. Van de Walle, C. G. & Neugebauer, J. First-principles calculations for defects and impurities: Applications to III-nitrides. *J. Appl. Phys.* **95**, 3851-3879 (2004).
8. Zhao, M., Chang, C., Xiao, Y. & Zhao, L.-D. High performance of n-type $(PbS)_{1-x-y}(PbSe)_x(PbTe)_y$ thermoelectric materials. *J. Alloys Compd.* **744**, 769-777 (2018).
9. Luo, Z.-Z. *et al.* Valence Disproportionation of GeS in the PbS Matrix Forms $Pb_5Ge_5S_{12}$ Inclusions with Conduction Band Alignment Leading to High n-Type Thermoelectric Performance. *J. Am. Chem. Soc* **144**, 7402-7413 (2022).
10. Luo, Z.-Z. *et al.* Enhancement of Thermoelectric Performance for n-Type PbS through Synergy of Gap State and Fermi Level Pinning. *J. Am. Chem. Soc* **141**, 6403-6412 (2019).
11. Hou, Z. *et al.* Contrasting Thermoelectric Transport Properties of n-Type PbS Induced by Adding Ni and Zn. *ACS Appl. Energy Mater.* **4**, 6284-6289 (2021).
12. Zhao, M. *et al.* Investigations on distinct thermoelectric transport behaviors of Cu in n-type PbS. *J. Alloys Compd.* **781**, 820-830 (2019).
13. Cheng, R. *et al.* Bridging the miscibility gap towards higher thermoelectric performance of PbS. *Acta Mater.* **220**, 117337 (2021).
14. Wang, S. *et al.* Fine Tuning of Defects Enables High Carrier Mobility and Enhanced Thermoelectric Performance of n-Type PbTe. *Chem. Mater.* **35**, 755-763 (2023).
15. Zhu, Y. *et al.* Large Mobility Enables Higher Thermoelectric Cooling and Power Generation Performance in n-type $AgPb_{18+x}SbTe_{20}$ Crystals. *J. Am. Chem. Soc.* **145**, 24931-24939 (2023).
16. Goodman, P. & Moodie, A. F. J. A. C. S. A. Numerical evaluations of N‐beam wave functions in electron scattering by the multi‐slice method. *Acta Crystallogr. A* **30**, 280-290 (1974).
17. Koch, C. T. *Determination of core structure periodicity and point defect density along dislocations*, Arizona State University, (2002).
